# Mask-Assisted Deposition of Ti on Cyclic Olefin Copolymer Foil by Pulsed Laser Deposition

**DOI:** 10.3390/mi14071298

**Published:** 2023-06-24

**Authors:** Mariapompea Cutroneo, Letteria Silipigni, Anna Mackova, Petr Malinsky, Romana Miksova, Vaclav Holy, Jan Maly, Marcel Stofik, Petr Aubrecht, Dominik Fajstavr, Petr Slepicka, Lorenzo Torrisi

**Affiliations:** 1 Nuclear Physics Institute of CAS, v.v.i., Husinec-Řež 130, 250 68 Řež, Czech Republic; 2 Department MIFT, Messina University, V. le F.S. d’Alcontres 31, S. Agata, 98166 Messina, Italy; 3 Department of Physics, Faculty of Science, J. E. Purkinje University, Pasteurova 3544/1, 400 96 Ústí nad Labem, Czech Republic; 4 Department of Condensed Matter Physics, Faculty of Mathematics and Physics, Charles University, Ke Karlovu 5, 121 16 Praha, Czech Republic; 5 Centre of Nanomaterials and Biotechnology, Faculty of Science, Jan Evangelista Purkyně University in Ústí nad Labem, 400 96 Ústí nad Labem, Czech Republic; 6 Department of Solid State Engineering, University of Chemistry and Technology Prague, 166 28 Prague, Czech Republic

**Keywords:** pulsed laser deposition, cyclic olefin copolymer, optical spectroscopy, Z-potential, SEM-EDX, AFM

## Abstract

Cyclic olefin copolymer (COC) is a novel type of thermoplastic polymer gaining the attention of the scientific community in electronic, optoelectronic, biomedicine and packaging applications. Despite the benefits in the use of COC such as undoubted optical transparency, chemical stability, a good water–vapor barrier and biocompatibility, its original hydrophobicity restricts its wider applicability and optimization of its performances. Presently, we report on the optical and morphological properties of the films of COC covered with Ti in selected areas. The layer of Ti on COC was deposited by pulsed lased deposition processing. The Ti/COC film was characterized by UV–Vis spectroscopy indicating that its transmittance in the visible region decreased by about 20% with respect to the pristine polymer. The quality of the deposited Ti was assessed with the morphology by scanning electron (SEM) and atomic force microscopies (AFM). The modification of the wettability was observed by the sessile drop method indicating a reduction of the native hydrophilicity.

## 1. Introduction

A polymer can be envisioned as a large molecule named macromolecule composed of many repeated sub-units commonly appointed as monomers. Depending on the type of these monomers the polymers can be broadly classified as organic if their skeletal structure includes carbon such as in Polyethylene or Poly(methyl methacrylate), to name a few, or inorganic if it is not included like in the polydimethylsiloxane [1].

The uncommon properties exhibited by polymers such as their flexible, lightweight, low-cost and optical properties make them a valuable part of our life not only as consumer products but also in innovative technology [2], fundamental and [3] material sciences [4].

Evidence of the growing interest in the synthesis and modification of organic polymers is readily revealed by the increasing development of polymer light-emitting diodes (PLEDs) [5], electro active polymers (EAPs) for the realization of electrically stimulated cells (muscle tissue) or synthetic tissues [6] and microfluidic/organ-on-chip devices for cell cultivations, migration assays and organ simulations [4].

Plastic microfluidic devices are a promising alternative to devices fabricated from glass, quartz or silicon because they are less expensive and more easily produced in large quantities.

Most of the synthetic polymers reveal a chemical inertness and high hydrophobicity which are not always desirable features. For instance, in microfluid application, hydrophobic surfaces restrict the smooth liquid injection and flow control inside microfluidic channels; in growth and transport of cells, the chemical inertness strengthens the lack of functional surface groups for immobilizing biomolecules [7].

Across the family of organic polymers, the cyclic olefin copolymer is a new thermoplastic material with glass-like optical transparency, low absorbance to moisture, high moldability, high resistance to organic solvent, high glass transition temperature, low autofluorescence and biocompatibility gaining the foremost role for several applications.

Its optical transparency and its resistance to water vapor and air make it feasible choice as a protective barrier film of devices, during the routinely innumerable operational cycles, for the prolongation of their duration [8]; its transmittance from the visible up to the near UV spectrum, higher than that of Polystyrene (PS) and (PC) and comparable to that of polymethylmethacrylate (PMMA), in addition to its low birefringence, low haze, lower density and diminished brittleness, make it ideal to replace glassy optical components; its high resistivity and dissipation factor, lower than that of PS and polypropylene (PP), are promising for the fabrication of thin film capacitors [9]. Metallized COC films reveal mirror properties lessening the permeability to light and increasing twofold the moisture barrier property of high density polyethylene (PE) and seven times that of unoriented PP and ten times that of polyvinyl chloride (PVC) [9].

**Figure 1:**
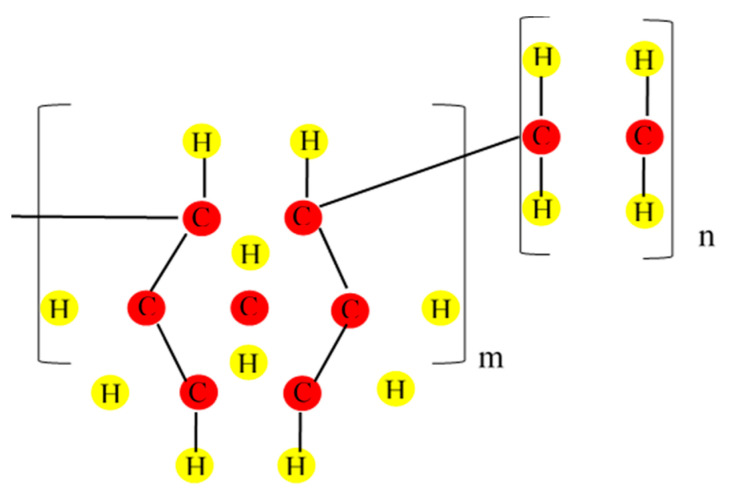
Sketch of the structure of the cyclic olefin copolymers.

The chronicle of COCs starts in the 1950s, while in 1976 is dated the discovery of a new class of catalyst “metallocene complex” for ethylene polymerization discovered by Kaminsky and Sinn. Their catalysts, besides several modifications, promoted the connection between cyclic and linear olefins leading to the synthesis of ethylene-α olefin co-polymers. The benefit of these catalysts is the enabling of large-scale copolymerization of ethylene and norbornene and a high degree of uniformity in its composition [10]. In Figure 1 is displayed the COC chain consisting of the monomers of ethylene and norbornene identified by the subscripts n and m indices, respectively.

A lot of cyclic monomers can be used to prepare COC; however, all of them are derivatives of norbornene or dicyclopentadiene with close properties.

Norbornene is a bicyclic olefin as it consists of a bridged six-membered ring with a double bond on one side. The bridged ring adds extra strain on the double bond, making it highly reactive, promoting the modification of the basic norbornene molecule or its incorporation into bigger molecules, such as COC or other polymers. The rigid structure of the norbornene inhibits the crystallization, making the COC amorphous, mechanically stiff, and optically transparent with a high moisture barrier. In addition, the high glass-transition temperature (Tg) in COC can range between 75 °C and 170 °C because it is linearly related to the content of the cyclic monomer and to the chemical structure of COC [11].

However, COC is hydrophobic, and microchannels made from it are likely to adsorb some analytes, leading to degradation of resolution in separations, sample loss and inaccurate quantitative analyses. To minimize adsorption of processed compounds, analytes demand the modification of the COC wettability [12].

Intensive research has been conducted for the preparation of desirable hydrophilic surfaces where there is a lower amount of blood plasma proteins adsorbed on it in comparison with hydrophobic surfaces [13].

In this view, the acquired hydrophilicity of COC surface displaying a contact angle of 79.6° when coated with Ti by pulsed laser deposition (PLD) can be of interest for biological application.

Previous papers cover techniques to lessen the hydrophobicity of the native COC include plasma treatment [14] or ultraviolet (UV)/ozone oxidation [15] even with disclosed surface damage and degradation. A technique which emerges for its reliability in the fabrication of films of selected material on matrices suitable for optical waveguide and microfabrication techniques [16] could be the pulsed laser deposition [17] which has proven its effectiveness in the transfer or the deposition of all solid material/compounds, with accurate and precise control of thickness and stoichiometry.

The excellent properties of titanium, such as high corrosion resistance, biocompatibility and a higher strength-to-weight ratio than steel, make it a promising material for the production of functional coatings, as active film for packaging [18] and in the manufacture of metal components, particularly in the aerospace and medical fields.

In this work, the formation of a thin layer of Ti on a COC is obtained by pulsed laser deposition which can be considered a low-temperature deposition processing able to change the surface properties of a material with complex engineered topography maintaining the bulk attributes. The manufacturing of COC/Ti foils could be beneficial for their successful interface with biomolecules, cells [19] and tissues such as catheters, orthopedic load-bearing implants and coronary stents.

When thin functionalized films are prepared, precise control of their thickness and composition represents two important key aspects, significantly affecting the film properties, its possible applications and characterizations. The morphology, wettability and composition of the deposited Ti inside and outside the pattern manufactured on COC film have been investigated to validate the suitability of the processing to improve its performance in biomedical applications.

The applied methods, the adopted conditions and the material used as substrate and deposited layer are crucial to obtain controlled composition, homogenous surface and stability in the deposited physiological environments.

## 2. Materials and Methods

### 2.1. Preparation of COC Film

The micro resist technology GmbH [20] provided the formulation mr-IT85-5.0 copolymer for the fabrication of a COC film with thickness of 10 μm. One claimed advantage in the use of this product seems to be the appropriate concentration of the polymer and the solvent is less toxic than others.

The first step in the manufacturing of the COC film consists of the cleaning of a silicon wafer with acetone and isopropanol and then its dehydration at 200 °C for 15 min on a hotplate. Polydimethylsiloxane (PDMS) supplied from Dow Corning Corp. is an elastomer obtained mixing of a 10:1 ratio of the silicon base and the curing, respectively, degassed for 45–60 min under vacuum in a desiccator. This solution of PDMS has been uniformly distributed on the previously cleaned wafers by spin coater according to the indications of the supplier [20], cured on the hotplate at 95 °C for 30 min and placed inside a plasma etcher (ICP-RIE plasma etcher SI 500, SENTECH Instruments GmbH, Berlin, Germany) for 30 s to activate the PDMS surface by oxygen plasma. The detail of the working conditions of the ICP (inductively coupled plasma) has been specified in the previous work [21]. After this processing, the wafers with PDMS activated by plasma were ready to be used for thin cyclic olefin copolymer (COC) layer deposition.

The formulation mr-I T85-5.0 copolymer has been spin-coated on PDMS (LabClaster, SÜSS MicroTec, Garching, Germany) at 500 rpm for 10 s and then 3000 rpm (500 rpm/s ramp) for 30 s and hot baked at 120 °C for 2 min. The deposited layer of COC 10 μm thick was then peeled off and cut in pieces of desired dimensions to fit into different characterization tools.

### 2.2. Pulsed Laser Deposition Mechanism

Pulsed laser deposition (PLD) is a technique routinely adopted for the fabrication of thin film deposited on a matrix by the ablation of a solid target irradiated by laser pulses in high vacuum.

The characteristics of the laser generated plasma strongly depend on the incident laser fluence (i.e., laser pulsed energy/spot area), wavelength and pulse duration, by the irradiation conditions (incidence angle, laser focalization and irradiation environment) and target properties (density, electrical and thermal conductivity and surface morphology). The interaction between these parameters is complex and, in special conditions, may induce the formation of nanoparticles and nanostructures with different composition, shape, size and distribution.

A laser beam is focused by a lens on the target placed into a vacuum chamber. In this low-pressure background, the photons interact with the solid target transferring the energy to the material mainly via the electrons of the target atoms. The penetration depth, depending on the absorption coefficient at the laser wavelength, can range between tens of nanometers to tens of microns or more, depending on the laser pulse energy. The smaller the absorption coefficient, the larger the penetration depth [22]. The transferred laser energy can excite both free and bound electrons; some electrons may be excited from the valence to the conduction band; part of the bound electrons can immediately be freed (i.e., nonthermal mechanism), and part can be excited to higher energy states. The energy of the excited electrons is transferred to the lattice through electron–phonon coupling in the order of 10^−12^ s, starting the photo-thermal ablative processes (for IR laser incident in metallic targets) [23]. Photochemical processes are instead induced by UV laser producing photoablation with chemical bond breaking of the target molecules [24].

The laser pulse generally has a duration of ns or sub-ns, and its high intensity induces fast processes of ionization and evaporation with plasma production at high temperature and density with a duration comparable with the laser pulse. Plasma is a non-equilibrium state and expands at high velocity and emits radiation due to its high temperature and energetic particles accelerated by the inner developed electric fields. 

Generally, at short laser wavelengths, in the UV region, low density plasma, ions and small particles are expelled during the laser–matter interaction. The photon energy of the laser light, without heating effects, induces bond breaking of the atomic lattice, ionization and molecular scissions, followed by ejection of atoms and ions [22]. On the contrary, at high laser wavelengths, in the IR region, high yields of removed ions and particles, with high energy, are ejected from the laser irradiated target, and thermal effect and the formation of particulate and droplets [23] are induced. Such droplets may have a size from nm up to micron scale.

Another important parameter is the laser energy which must be higher than the ablation threshold [24] to remove the material from the solid target. At laser fluence slightly above the threshold, low ablation and low deposition rates can be generated, while increasing the laser fluence an almost linear increase of the ablation rate is induced [23].

### 2.3. Pulsed Laser Deposition Processing

**Figure 2:**
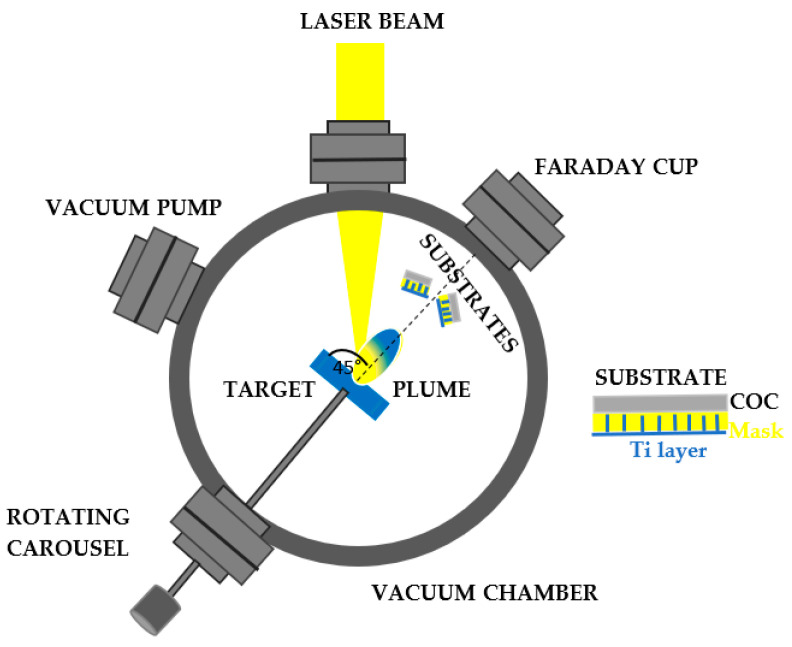
Schematic illustration of the pulsed laser deposition system.

**Figure 3:**
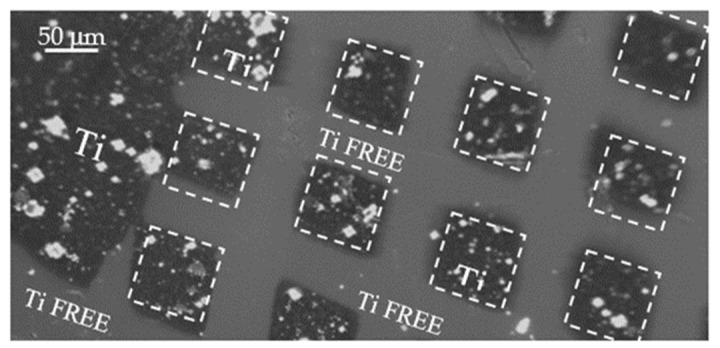
Optical microscope image of the pattern realized on COC foil.

The Ti deposition of thin film on the surface of a COC matrix is carried out through the direct IR laser irradiation in repetition rate of a solid Ti target placed in the vacuum. Above the ablation threshold of the solid target, ionization is induced, and a bright-colored plasma is formed. Such a plume consists of electrons, ions, clusters and exhibits a stoichiometry similar to that of the laser-irradiated material. Results that the particles ejected at high energy from the laser generated plasma are collected on a substrate placed near to the solid target, as observed in Figure 2. The COC was covered with a metallic mask with a thickness of about 1 mm and designed with an array of squares with a lateral size of about 80 μm × 80 μm (see Figure 3).

A Q-switching Nd:YAG laser system purchased from Litron [25], operating at the IR wavelength, pulse duration of 5 ns, 1064 nm of wavelength and 10 Hz repetition rate, has been used to irradiate a Titanium (Ti) solid sample (99.99% purity) located in a chamber at a vacuum of 5.5 × 10^−5^ mbar [26]. Because a high energy laser and IR wavelengths are being used, a condensed phase of droplet can form on the deposited layer worsening its quality and properties. The incidence angle has been set at 45°. According to the literature, at a glancing angle of the incident radiation, atomic particles are generated from the plume and deposited on the substrate [27]. The laser energy of 600 mJ has been focused on a beam spot of 1 mm in diameter on the target. The layer of Ti has been produced hitting the solid target with 10,000 laser shots in repetition rate for 13 min. The laser fluence was about 60 J/cm^2^.

In addition, the surface roughness of the primary target to be laser irradiated has been polished to obtain a reduction of the droplet phase in the laser plume [28], and the target-substrate distance has been increased up to 5 cm since the decrease of the laser spot ellipticity leads to a reduction in the ablated phase. Typically, the density of the droplet phase can be calculated from the squared distance from the normal surface between the laser spot and substrate [28].

A COC film 10 μm thick has been covered with a metallic (Ni) mask of about 1 mm thick and placed about parallel to the target surface and at 5 cm from the main target as shown in Figure 2. Behind the substrates, at a distance of 1 m from the target, the energy of the laser generated ions has been monitored by a Faraday cup (FC) [29] in time of flight (TOF) configuration and recorded with a fast storage oscilloscope (Tektronix, Beaverton, OR, USA).

### 2.4. Optical Characterization (UV–Vis)

The UV-Vis system, consisting of an AvaSpec-2048 spectrometer (Germany) provided with an UB-600 lines/mm grating and working at wavelengths between 400 nm to 650 nm, has been employed to monitor the optical modification induced in the COC foil after the deposition of Ti by PLD. The Halogen/deuterium light has been used as source to illuminate, through an optical fiber, the pristine and deposited COC on a surface of 2 mm^2^ in transmission mode. The light is guided in front of the sample at 0° and a second fiber, optically connected to the spectrometer, has been located behind the sample to measure the transmitted light.

### 2.5. Wettability Measurements

The wettability of the pristine and the deposited COC has been examined by the sessile drop method. A volume of 3 µL of deionized water has been dropped on the surface of the foils, and the formed contact angle displayed on the optical microscopy screen has been measured by dedicated software [30]. The measure of this angle has been acquired in different areas of the sample surface to obtain the average standard deviation value at room temperature, 1 bar and 50% air humidity. 

### 2.6. Scanning Electron (SEM) and Atomic Force (AFM) Microscopies

The topographical and morphological alteration of the surface and the roughness of the pristine and Ti-covered COC have been monitored by scanning electron microscopy (SEM using a LYRA3 GMU (Tescan, Brno, Czech Republic) operating at an acceleration voltage of 5 kV and in the secondary-electron mode. The view fields of the analyzed areas were 15 μm and 4.77 μm. The energy dispersive X-ray fluorescence (EDX) coupled to SEM provides the maps collected from the characteristic Kα lines in C (about 277 eV), O (around 525 eV) and Ti (1.510 keV) elements on the surface and on the cross-sections of the thin foils.

A Dimension ICON (Bruker Corp., Billerica, MA, USA) equipped with the ScanAsyst mode in air has been used to carry out AFM images. Areas of 3 × 3 μm^2^ of pristine and deposited COC has been captured at 1 s exposure time and processed by NanoScope Analysis software for the evaluation of the surface average roughness (Ra) and the mean roughness parameter (RMS).

### 2.7. X-ray Diffraction on Cyclic Olefin Copolymer Deposited with Ti

The crystalline phase of COC pristine and when it is deposited with Ti by pulser laser deposition has been monitored by X-ray diffraction (XRD) using a diffractometer PANalytical Empyrean (Malvern, UK) operating at 45 kV and 40 mA with a Cu-target tube, using a high-definition Bragg-Brentano setup; the samples have been placed on a diffractionless Si plate. Symmetric 2θ/ω XRD curves have been collected in the (10–110)° region in 2θ.

## 3. Results

Although the theoretical ablation threshold of Ti is of about 0.6 J/cm^2^ [24], a value 100 times higher has been selected to remove more material in a shorter time. It is commonly accepted that when the laser fluence overcomes the vaporization threshold the shape of the crater changes and its depth is higher than at the threshold value [31]. However, the use of the rotating carrousel used in the present experiment enables the laser irradiation of fresh surfaces, and it can avoid alteration of the laser energy density due to the irradiation of the deeper crater.

The surface pattern consisting of localized squares with a size of about 80 μm × 80 μm has been replicated covering the pristine COC with a metallic (Ni) mask, of about 1 mm thick, full size 20 mm × 30 mm. In Figure 3, an optical image 10x magnified pointed out the surface of the COC after the pulsed laser deposition of Ti. 

It is possible to distinguish the Ti free areas and the ones deposited with Ti marked by dashed white lines. Figure 3 displays a detail of the pattern realized by laser deposition of Ti ions.

**Figure 4:**
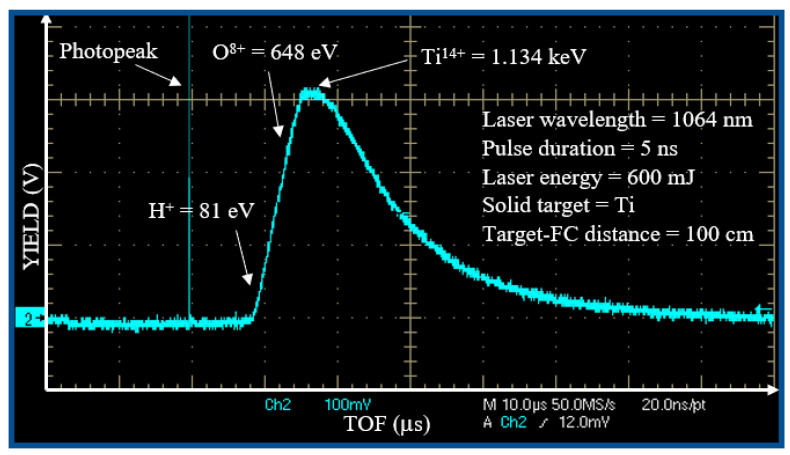
Spectrum in TOF obtained from a Faraday cup for a target of Ti irradiated by laser.

The deposition fulfilled on the surface of the COC foil is due to the products of the laser–matter interactions [32] consisting of electrons, ions, photons, clusters and neutrals. The primary contribution is attributable to the laser generated multi-energy and multi-species ions. Typically, high energy ions can reach high depths inducing implantation while low energy ions can be deposited at shallow depths in the substrate. Since the ion energy is a key factor affecting the structural, chemical and physical modifications in the materials, it is conventionally monitored using different detectors such as SiC, the Thomson Parabola spectrometer [33] and the Faraday cup (FC) in the Time-Of-Flight (TOF) configuration [3]. Figure 4 shows a typical spectrum collected when the Ti target has been irradiated by a single laser pulse with an energy of 600 m J focused on 0.5 mm^2^ spot size. The first sharp, fast and narrow peak is the photopeak due to the photoelectric effect, with a width of about 10 ns indicating the plasma duration. The wide peak with a long tail is attributable to the detection of ions. The faster ions are protons (H^+^), followed by the oxygen (O^n+^) ions of the Ti surface and by the titanium (Ti^n+^) ions of the target bulk, in agreement with the literature [32].

The intense photopeak is followed by a large, structured peak extending from 8 to about 50 μs.

This structured ion peak detection is due to the convolution of the signal of the fast protons followed by contaminants as O ions (from the surface of the titanium target) and Ti ions at different charge states.

The TOF approach permits calculation of the ion energy. The maximum proton energy is about 81 eV; that of oxygen ions is about 648 eV; and that of titanium ions is about 1134 eV. Such result indicates that the plasma ion acceleration is of about 81 eV/charge state; thus, O ions are ionized from O+ up to O^8+^ (the most accelerated ones), and the Ti ions are ionized from Ti^+^ up to Ti^14+^ (the most accelerated ones).

Because the ionization potential of O^7+^ is 739 eV and that of Ti^13+^ is 787 eV [34], the evaluated maximum electron energy is of about 790 eV.

Assuming a Boltzmann electron energy distribution, the mean electron energy is about one-fifth of the maximum value, i.e., <E> ~ 158 eV, at which corresponds a mean electron temperature of about:

kT = (2/3) <E> ~ 105 eV(1)

At the evaluated Ti ion energy, between 1.134 keV and the peak tail, at about 80 eV, their deposition in the solid COC polymer induces dominant nuclear-stopping power affecting the roughness of the COC surface through degradation and sputtering effects. Such effects are also produced in the Ni metallic mask. SRIM code [35] permits the evaluation of a sputtering yield of Ti ions at 1 keV energy in Ni (mask) and C (polymer) of about 2.9 atoms/ion and 0.15 atoms/ion, respectively.

The evaluated Ti thin film thickness is about 100 nm in the used irradiation time, in agreement with the literature reporting a laser ablation yield of about 0.4 μg/pulse at a laser fluence of about 60 J/cm^2^. Of course, the Ti film thickness could be controlled by the irradiation time using the 10 Hz repletion rate and the same laser irradiation parameters described in this investigation.

**Figure 5:**
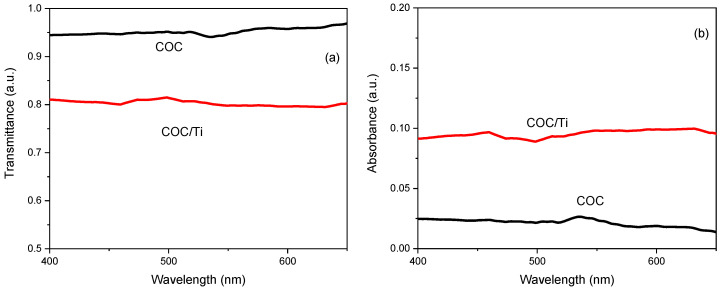
Transmittance (**a**) and absorbance (**b**) for pristine COC and for COC deposited with Ti ions.

The optical modification induced in the COC foil after the PLD processing has been estimated through the comparison of the original transmittance (see Figure 5a) and absorbance (see Figure 5b) in pristine and Ti-covered COC.

In Figure 5a is displayed the high transmittance of the pristine COC in accordance with its high native glassy-transparency and the optical response of the Ti/COC showing a decrease of about 16%. The comparison between the absorbance in the pristine and Ti/COC shows an increase of about 3%. The spectra viewed in Figure 5 indicate small changes of the optical features due to the presence of the Ti deposit which seems to be responsible for the alteration of the roughness and composition of the pristine COC surface.

**Figure 6:**
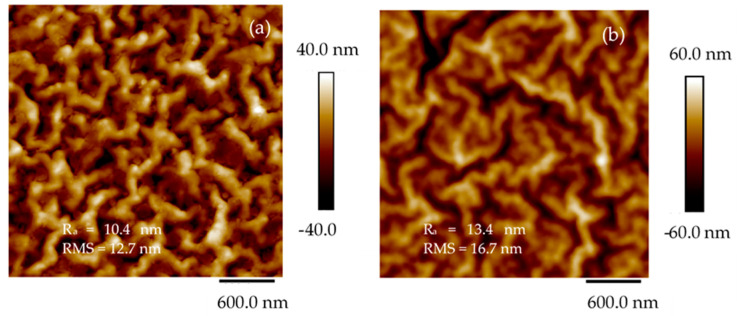
AFM images of the pristine COC (**a**) and of the Ti deposited COC (**b**).

To assess the quality of the pattern reproduced on COC through pulsed laser deposition of Ti, AFM and SEM analyses have been conducted. In Figure 6 are the 2D images of the pristine COC (see Figure 6a) and of the areas inside the deposited pattern (see Figure 6b) alongside the average surface roughness (Ra) and mean roughness parameter (RMS).

The NanoScope Analysis software pointed out the difference between the investigated area of the sample and the rough surface of the same sample which increases from 5.55% in pristine COC to 6.06% in COC deposited by PLD. This output highlights the increase of the surface roughness in agreement with the RMS rising from 12.7 nm in pristine COC to 16.7 nm in the deposited COC, respectively. The structures deposited on COC by PLD, exhibiting a size lower than 16.7 nm, are ascribable to the laser irradiation of the solid target at a glancing angle according to the literature [27].

The AFM data, derived from an average of four scans from different areas on the films, yield an RMS roughness of 12.7 nm (see Figure 6a) on the pristine COC and 16.7 nm (see Figure 6b) on the Ti/COC deposited by laser ablation.

The quality and the elemental composition of the pristine polymer surface, of the COC surface directly exposed to Ti ions deposition and of the COC surface covered by the metallic mask, have been investigated using 5 keV SEM and electron X-ray fluorescence (EDX). Table 1 lists the elemental compositions of pristine COC and COC deposited with Ti ions inside the squares and below the grid. The composition includes the main component of C at 99.0 ats% in accordance with its formulation but with the detection of hydrogen (which should be the second component) being unfeasible by EDX. The O concentration of about 9.40 ats% in deposited COC is probably imputable to the oxygen contaminants on the surface of the Ti target used to partially cover the COC surface.

**Figure 7:**
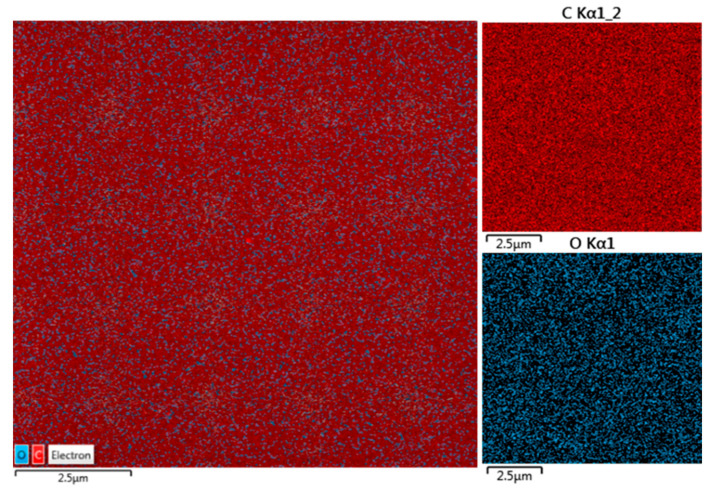
SEM-EDX images of pristine COC.

The evaluated concentration of Ti deposited inside the squared patterns on COC foil is of about 5.27 ats%. The maps shown in Figure 7 illustrate the results listed in Table 1. In the insert in Figure 7, the maps referring to C and O Kα lines have been provided by the energy dispersive X-ray coupled to SEM.

The uniformity of the red color covering all of the pristine COC foil seems to confirm the absence of heavy elements which is evidence of its purity.

**Figure 8:**
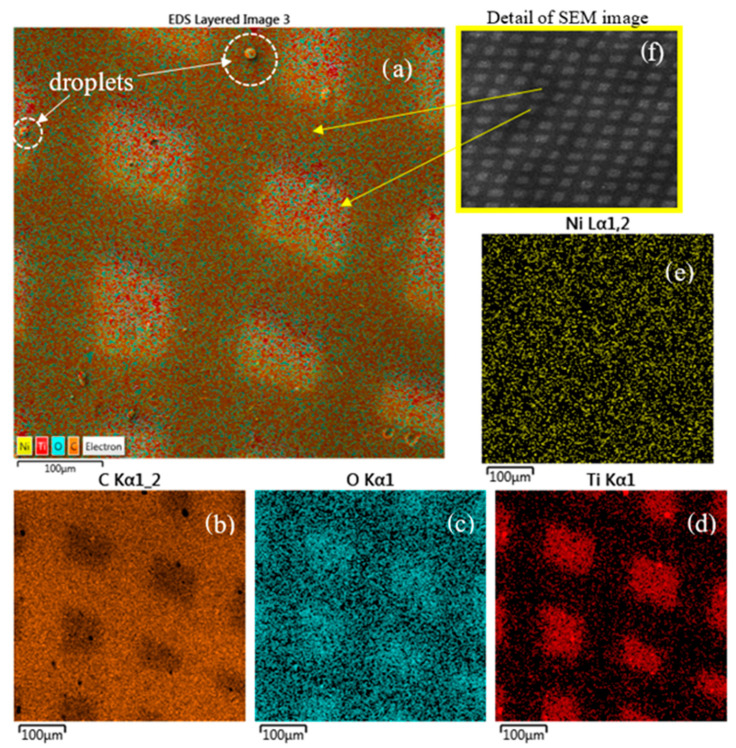
Elemental maps of the surface (**a**–**e**) of the COC deposited with Ti and a more detailed SEM image (**f**).

The compositional analysis by elemental mapping shown in Figure 8 indicates the presence of C (see Figure 8b), O (see Figure 8c), Ti (see Figure 8d), Ni (see Figure 8e) and Kα lines provided by the energy dispersive X-ray coupled to SEM on the surface of the Ti/COC film.

Moreover, the detection of Ni by SEM-EDX is related to the composition of the mask used during the processing and the Ti ion sputtering effect on it. SRIM demonstrates that the sputtering yield of 0.6 keV Ti ion in Ni is 2 atoms/ion [35]. Moreover, in Table 1 is listed the composition of the first superficial layers of the COC covered by the Ni mask indicating a concentration of about 0.70 wt% of Ni and 0.01 wt% of Ti. This result suggests that the laser plasma pulse generation at 10 Hz repetition rate for deposition times of about 13 min induces both thermal effects and ion-sputtering effects on the mask and on the polymer. The thermal effects heat the mask and the polymer while the sputtering effects are responsible for the transfer of Ni atoms to the below polymer surface. The thermal heating favors the Ni and Ti infiltration and diffusion under the mask, in the mask–COC interface.

The quite uniform red color distribution on the map confirms the Ti deposition inside the squared patterns on COC. The presence of the O displayed by blue color is assignable to the impurities on the surface of the Ti target from which the Ti ions/clusters and the droplets have been released during the laser deposition processing.

The circular structures located on the surface of the deposited COC and marked by dashed white color are a doplets phase of Ti emitted from the laser plume and deposited on the surface. A detailed SEM image of the pattern with a wide field of 4.77 mm and 44 X magnification is shown in Figure 8e for a better visualization of the pattern displayed in the SEM-EDX (see Figure 8a–e).

**Figure 9:**
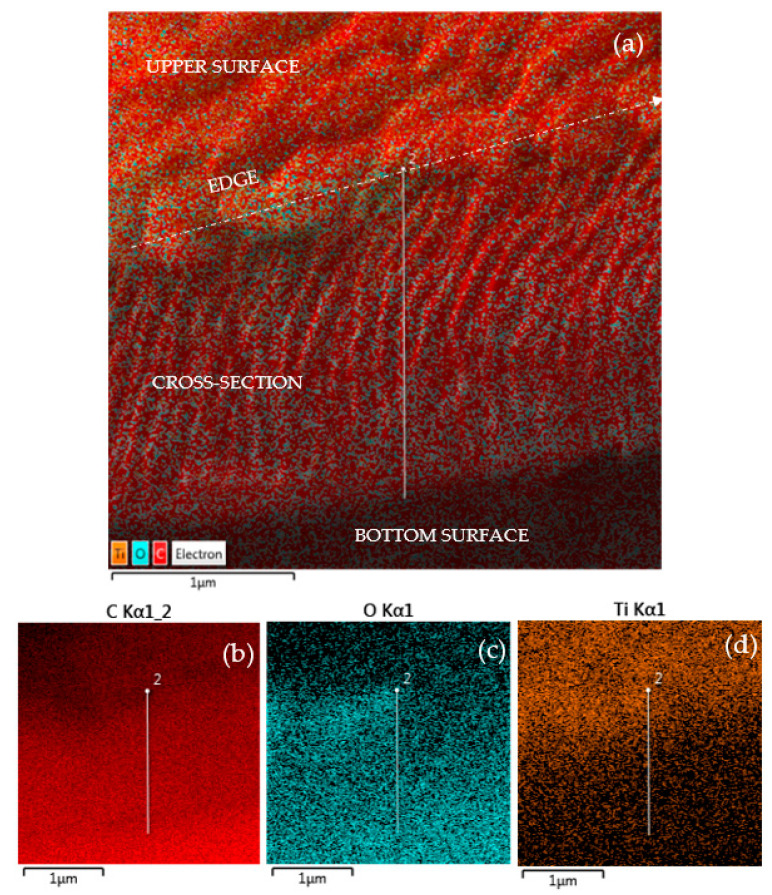
Maps of the cross-section of the COC foil deposited with Ti by PLD.

COC foil deposited with Ti has been transversally cut and placed at a planar angle for a simultaneous display of the surface and cross-section. In Figure 9 have been marked, in white color, the upper surface, the cross section and the bottom surface to assist the visualization. Figure 9 shows the maps indicating the presence of C (see Figure 9b), O (see Figure 9c) and Ti (see Figure 9d) Kα lines provided by the energy dispersive X-ray coupled to SEM. It seems that both C and O are more pronounced inside the cross-section, whereas Ti is distinct at the surface and slightly fades in the first layers of the cross-section due to the nuclear stopping power of the low energy Ti ions in the COC foil.

In the elemental maps shown in Figure 8c,d, there appears to be overlap inside the deposited squares of both O and Ti. However, the cross-sectional analysis shows a strong presence of O inside the COC foil, maybe due to the manufacturing methodology of the COC.

**Figure 10:**
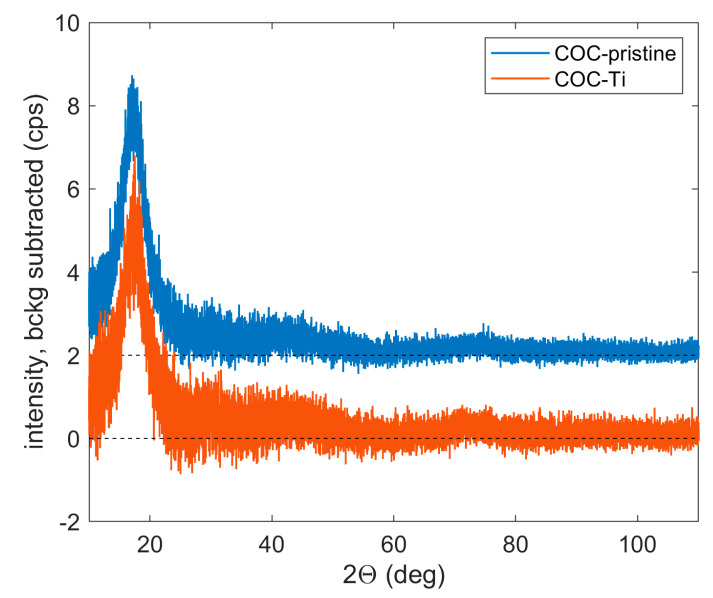
XRD curves of pristine COC and deposited with Ti. From the measured intensities, the diffuse background from the Si sample holder was subtracted; the blue curve is shifted upwards for clarity.

The XRD curves displayed in Figure 10 are referred to as the pristine COC and the COC deposited with a very thin layer of Ti by PLD. The curves of both samples are almost identical; the broad peak at approx. 17.0 ° can be ascribed to amorphous COC. The absence of the Ti signal can be explained by a very low thickness of the deposited layer.

**Figure 11:**
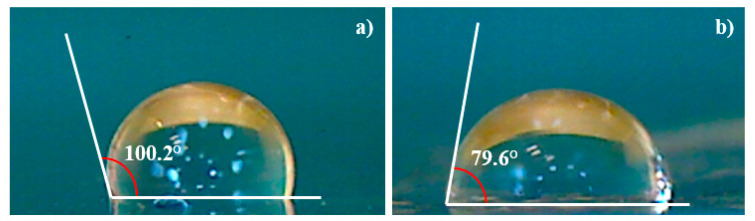
Contact angle for virgin COC (**a**) and for COC deposited with Ti ions (**b**).

The surface wettability has been investigated through the measure of the contact angle on the pristine and deposited COC. The degree of phase separation has been determined from the angle formed at the liquid/solid and the liquid/air interfaces when a drop of deionized water contacts the surface of the samples as shown in Figure 11.

The decrease of the contact angle from 100.2° in pristine COC (see Figure 11a) to 79.6° in Ti deposited COC (see Figure 11b) suggests that the increased wettability may be affected by the presence of structures with sizes bigger than the 16.7 nm as displayed in AFM images. 

These results showed that deposited titanium on COC increased the hydrophilicity resulting in a prospective improvement in cell growth and interaction, in agreement with the literature [36].

The increase in the surface roughness might turn the surface from hydrophobic to hydrophilic.

The increase of the wetting ability of the Ti/COC could have been predicted because the wetting angle in pure Ti surface is about 70° [37]. It can be concluded from this result that the COC hydrophobicity decreased by the presence of a non-uniform Ti layer. Increasing the laser deposition time, the formed Ti layer could be thicker, more uniform and probably more beneficial for the growth of cells on Ti/COC film leaving unchanged the native properties of COC.

## 4. Discussion

In this study, cyclic olefin copolymer (COC) foil has been used as substrate to be covered by Ti thin film through a mask produced by pulsed laser deposition technique (PLD) for prospective biomedical applications. The pulsed laser deposition is a well-established technique based on three steps: laser ablation of target, plasma plume expansion and layer growth on the substrate. The interplay of the laser wavelength and energy, pulse duration, environmental background, target-substrate geometry and composition are crucial in the accomplishment of optimized deposition.

The laser energy has been selected to ensure congruent and sufficient ablation to produce the required plasma species. When the plasma propagates and expands reacting with the background pressure, the species ejected from the plasma plume having the same stoichiometry of the direct laser irradiated target formed a Ti coating on the substrate surface. 

The kinetic energies of the species generated in the plasma, strongly related to the laser energy and laser wavelength by the relation Iλ^2^, have a direct effect on the adherence of the film deposition. The ion energy is a key parameter affecting the structural, chemical and physical modifications in the materials in view of the laser-generated multi-species and multi-energy. The high-energy ions enable implantation up to high depths while low-energy ions deposition on the surface of the substrate. 

Thermal contributions are observed when an IR laser with a pulse duration sufficiently long (5 ns), used in repetition rate for high exposition times, is employed in pulse laser repetition (PLD) mode. The high temperature of the produced plasma may heat the substrates on which the ablated material is deposited [23]. The greater energy deposited in the metallic mask during exposure results in mask heating and its instabilities, due to heat expansion, stress and ion implantation [38]. Moreover, the high energy of deposited ions may induce mask and substrates ion sputtering of their surfaces, with possible damaging effects.

The selected IR laser wavelength promoted the ablation rate while the damage induced by the droplets phase emitted from the laser plume and deposited on the COC surface has been partially minimized by the high thermal conductivity of the COC. 

The linear distance between the target and the substrate has been increased generating a plasma expansion distance long enough to assist the splitting before the substrate is hit minimizing the substrate degradation. This large distance should support lower thickness deposition.

The high biocompatibility and mechanical strength of Ti and its alloys mean they are the most used materials for biomedical implants and surgical devices [13].

The observed reduction of the transmittance, if it were higher, could be a limit for biological application. However, it could be an asset for the manufacturing of active films and coatings for food packaging, supporting the reduction of the visible component of light without hindering the identification of the packed item. Foils of COC coated with Al, in view of their reflectivity properties, have shown its possible applicability to preservation of products which can be degraded, such as horticultural products [18], and it is known that Ti exhibits higher reflectivity than Al.

The surface wettability quantifies the interaction of a liquid with a solid surface, depending on the intermolecular interaction of the liquid–solid surface and the cohesive force between the liquid molecules. 

Conventionally, surfaces are denominated super-hydrophilic if the contact angle is ∼0, hydrophilic if it ranges between 0° and 90°, hydrophobic if it is higher than 90° and superhydrophobic if it is higher than 150°. 

The native hydrophobicity of COC could lead to the evaporation of water vapor through channel walls in microfluidic devices [39] and altering the stability in the concentration of the used aqueous solution. This changing of the experimental conditions is undesirable, especially in biological applications. On the other hand, the hydrophobicity of COC could be beneficial as it could successfully prevent its incorporation into the used solutions. The matching between the hydrophobicity of COC and the native hydrophilicity of Ti could lead to a material with proper wettability and a perspective for broader use, from cell cultivation to microfluidic devices.

The reduction of the observed contact angle in COC/Ti foil is linked to the increase in the surface roughness [37] which slightly increases from 10.4 nm to 13.4 nm as reported by AFM analysis.

The morphology and composition of the layer and of the microstructure formed on the Ti/COC surface have been assessed with the aid of SEM-EDX. The small amount of Ti of about 5.3% is attributable to the deposition time and to the large target to substrate distance, while the oxygen content of about 9.7% to the contaminants on the surface of Ti target desorbed during the direct laser irradiation. 

Depending on the application, to reduce the oxygen content ejected from the plasma and deposited on the COC foil, it is common to use a shutter located in front of the substrate to be deposited for the first laser pulses, as a means to prevent the formation of oxides.

The oxide on a metal surface [37] is desirable to improve the local hardening and wear resistance and, due to the bio-inertness of titanium oxides, can affect the adhesion properties [40,41].

On the other side, the formation of oxide on titanium can engender rough and pitting corrosion. This can be overcome through the binding of the implant to the bone, particularly through the formation of a dense passive film, or in the case of medical implant, it is surrounded by fibrous tissue, which prevents the material from binding directly to the bone [42].

## 5. Conclusions

The paper is addressed to highlight the modifications of cyclic olefin copolymer (COC) foil by pulsed laser deposition to obtain, in vacuum, a thin layer of Ti on its surface using a metallic mask coverage.

The following characterizations of the Ti/COC samples have been accomplished to ensure the preservation of some COC properties and to modify others, as well for possible application in biomedical field. Biocompatibility, low-cost production, light weight, strength, ease of fabrication, thermal and chemical resistance, excellent optical properties and a high moisture barrier represent some of the original features of COC.

However, the natural hydrophobicity of COC may limit its use as microfluid because the adsorption of analytes as proteins affects the resolution of separation and the quantitative analyses; as substrate for the localized growth of cells it alters their adhesion, spreading, migration and proliferation. To turn the hydrophobicity into hydrophilicity, the pulsed laser deposition technique has been adopted manufacturing a COC foil covered with a thin layer of biocompatible titanium. SEM and SEM-EDX analyses indicate a good quality of the Ti coating film with the presence of only few droplets which, in the worst case, could seriously affect the foil feature but which can be minimized adopting proper laser setting parameters and geometry.

The measure of the contact angle on the deposited COC used to monitor and quantify the changes in surface hydrophilicity indicates a decrease from 100.1° to 79.6° leading to a more favorable spreading of liquid on the Ti/COC surface.

The obtained value is close to that of native Ti surfaces, suggesting that the changed wettability is attributable to the thin layer of Ti. The reversal from hydrophobicity to hydrophilicity is connected to the increase of the surface roughness probed by AFM images.

The results indicate the presence of nanostructures on the deposited COC surface with sizes of about 16.4 nm.

The work is in progress to assess the biocompatibility of the technique applied on COC, the applicability for localized functionalized areas of the foil for biomedical applications, such as for cellular growth in a controlled environment. The benefits of functionalized COC surfaces manufactured by Ti- PLD are the easy processing, high reproducibility and quality and low procedure costs.

## Figures and Tables

**Table 1:** Atomic composition of pristine and deposited COC by EDX analysis.

SAMPLE	ELEMENT				
	Concentration (ats%)				
	C	O	Ti	Ni	C/O
**Pristine COC**	99.0	1.0	-	-	99.0
**Inside the squares of COC deposited with Ti ions**	84.19	9.40	5.27	1.14	8.95
**Below the grid placed on COC during the deposition with Ti ions**	97.6	1.69	0.01	0.70	57.7
